# Rice-grounds

**Published:** 1844-10

**Authors:** 


*Rice-grounds.*—A committee of physicians, owners of rice-grounds, agriculturists, engineers, and chemists, was named in 1841, at the congress of Florence, to examine into the influence of rice-grounds on the health of those who cultivated them, and of those who resided near them. Their report, which was made at the congress at Lucca, is decidedly unfavorable. It stated that they are always unhealthy, even when established in well-ventilated localities, on dry ground, irrigated by water easily drawn off.

